# Implementation of a Computerized Decision Support System to Improve the Appropriateness of Antibiotic Therapy Using Local Microbiologic Data

**DOI:** 10.1155/2014/395434

**Published:** 2014-08-17

**Authors:** Manuel Rodriguez-Maresca, Antonio Sorlozano, Magnolia Grau, Rocio Rodriguez-Castaño, Andres Ruiz-Valverde, Jose Gutierrez-Fernandez

**Affiliations:** ^1^Department of Microbiology, Torrecardenas Hospital Complex, Hermandad de Donantes de Sangre s/n, 04009 Almeria, Spain; ^2^Department of Microbiology, School of Medicine, University of Granada, Avenida de Madrid 11, 18012 Granada, Spain; ^3^Intensive Care Unit, Torrecardenas Hospital Complex, Hermandad de Donantes de Sangre s/n, 04009 Almeria, Spain; ^4^Service of Microbiology, Virgen de las Nieves University Hospital, Avenida de las Fuerzas Armadas 2, 18014 Granada, Spain

## Abstract

A prospective quasi-experimental study was undertaken in 218 patients with suspicion of nosocomial infection hospitalized in a polyvalent ICU where a new electronic device (GERB) has been designed for antibiotic prescriptions. Two GERB-based applications were developed to provide local resistance maps (LRMs) and preliminary microbiological reports with therapeutic recommendation (PMRTRs). Both applications used the data in the Laboratory Information System of the Microbiology Department to report on the optimal empiric therapeutic option, based on the most likely susceptibility profile of the microorganisms potentially responsible for infection in patients and taking into account the local epidemiology of the hospital department/unit. LRMs were used for antibiotic prescription in 20.2% of the patients and PMRTRs in 78.2%, and active antibiotics against the finally identified bacteria were prescribed in 80.0% of the former group and 82.4% of the latter. When neither LMRs nor PMRTRs were considered for empiric treatment prescription, only around 40% of the antibiotics prescribed were active. Hence, the percentage appropriateness of the empiric antibiotic treatments was significantly higher when LRM or PMRTR guidelines were followed rather than other criteria. LRMs and PMRTRs applications are dynamic, highly accessible, and readily interpreted instruments that contribute to the appropriateness of empiric antibiotic treatments.

## 1. Introduction

Microorganism resistance to antibiotics changes over time and varies according to geographic area, hospital, or even hospital department [1, 2]. Various studies on severe infections in hospitalized patients, especially those in intensive care units (ICUs), have associated an inappropriate initial antibiotic treatment with increases in bacterial resistance, morbidity-mortality, hospital stay, and hospital costs [3–12]. Selection of the appropriate empiric antibiotic treatment requires knowledge of changes in the etiology of infectious processes and in antibiotic resistance patterns in each hospital area [13, 14].

The selection of empiric antibiotic therapy is generally based on updated clinical practice guidelines or therapeutic recommendations developed by expert groups from scientific societies [15–17], which must be adapted to the epidemiologic characteristics of each country or healthcare area [13, 18]. Once microbiological results are confirmed and the satisfactory clinical progression of patients is observed, it is recommended to deescalate antibiotic therapy when possible in accordance with the antibiograms of the identified bacteria [3, 6, 10, 11, 15, 19].

Hospital antibiograms are commonly used to monitor local trends in antimicrobial resistance and to prepare antibiotic policies for guiding targeted empiric therapy [20]. Thus, besides the identification and study of microorganism susceptibility, the microbiology laboratory has a role in periodically updating results for designing empiric antibiotic treatment guidelines adapted to the local microbial epidemiology [21, 22]. These guidelines should be based on the best available clinical evidence and on the resistance profiles in each healthcare setting [6, 18]. They need to be constantly updated, taking account of the clinical usefulness of treatments, the ease of their management, and consensus agreements among professionals [21]. Guidelines are considered to be more useful when defined and implemented by a multidisciplinary team and adequately disseminated and promoted, followed by evaluation of their acceptance and implementation [23, 24]. Ideally, these guidelines should be developed for each hospital department, and there is a particular need to avoid needless antibiotic administration for a suspected nosocomial infection in the ICU [25].

The objectives of our study were to design, develop, and implement a new computer application based on the local epidemiologic analysis of bacterial susceptibility to antibiotics and to assess the usefulness to physicians of the information that it offers for selecting the most appropriate antibiotic treatment in ICU patients with suspicion of nosocomial infection.

## 2. Patients and Methods

This study was conducted over a three-year period in a third-level 821-bed hospital,* Complejo Hospitalario Torrecardenas* (CHT), serving 350,000 inhabitants and eight primary care districts in the province of Almeria (southeast Spain).

### 2.1. Study Design

A prospective, quasi-experimental study was conducted in three stages between January 2008 and December 2010. During the first six months, a new computer-assisted program was developed for antibiotic selection. Between July and October 2008, four information sessions and two round table discussions were conducted with the specialized physicians and nursing staff of the ICU and Departments of Microbiology, Preventive Medicine, and Pharmacy of the hospital to promote the new program and train participating physicians in its application. Finally, between October 2008 and December 2010, the new system was implemented for antibiotic prescription in the ICU, the patients in the study were followed up, and the results were analyzed.

### 2.2. A Computer-Assisted Program for Antibiotic Selection

We developed an application, based on Microsoft.NET Framework with Visual C# and SQL, with Open DataBase Connectivity (ODBC) to the Laboratory Information System (LIS) of the Hospital Microbiology Department in order to provide real-time analysis and updating of data obtained from microbiological studies. The application, designated* Guía Electrónica de Resistencias Bacterianas* (GERB), was installed in a central server. The database is automatically updated and allows consultation of all antibiograms recorded in the LIS, extracting data according to different selection criteria (e.g., date or date interval, patients, samples, diagnoses, microorganisms isolated, antibiotics tested, and hospital departments), and creating graphics to facilitate interpretation and visualization on the computer screens in the network.

Two GERB-based computer applications were developed: local resistance maps (LRMs) and preliminary microbiological reports with therapeutic recommendation (PMRTRs). Both guidelines consider the epidemiology, the evidence-based best treatment options for the most prevalent bacterial pathogens, and the local-specific antibacterial pathogen susceptibility.

#### 2.2.1. Local Resistance Maps (LRMs)

These maps graphically depict the accumulated susceptibility data in the LIS for all bacteria identified in samples from ICU patients during the previous year and from the patients who have undergone antibiograms. The GERB system is used to create three types of LRM for the empiric treatment of* lower respiratory tract infections*, based on antibiograms for bacteria isolated from samples from the lower respiratory tract;* urinary tract infections*, based on antibiograms for bacteria isolated from urine samples; and* bacteremias*, based on antibiograms of bacteria isolated from blood cultures. The antibiotics included in the LRMs were selected by consensus among the ICU physicians; their inclusion required a minimum of 30* in vitro* assays. The percentage of bacteria susceptible to each antibiotic was depicted by using a color code: green: susceptible, yellow: intermediately susceptible, and red: resistant.

Physicians had ready access to the maps* via* touch screens located in the ICUs that were connected to the hospital intranet and automatically updated every 24 h, incorporating any new records entered into the GERB system.

#### 2.2.2. Preliminary Microbiological Reports with Therapeutic Recommendation (PMRTRs)

A PMRTR was issued when the microbiology laboratory reported a sample to be positive according to the culture results but before a definitive identification and antibiogram. The requesting ICU physician was informed about the microorganism genus or isolated microorganism or, when not available, the result of Gram staining and was given therapeutic recommendations. These named the antibiotics with highest activity against the microorganisms presumably involved and against the specific infectious disease of the patient, according to the information in the GERB, and they included the most favorable pharmacokinetics and pharmacodynamics properties according to the infection focus. This signed report was sent directly to a dedicated remote printer in the ICU.

### 2.3. Patients

Inclusion criteria for the patients in the polyvalent 24-bed ICU were as follows: (i)* suspicion of nosocomial infection*, defined as developing ≥48 hrs after ICU admission, in the lower respiratory tract, urinary tract, or blood (bacteremia), based on clinical symptoms and results of laboratory tests or radiologic exam. The focus was microbiologically defined as the lower respiratory tract or urinary tracts when the corresponding microbiological cultures were positive, regardless of the presence of bloodstream infection; bacteremia was defined by the isolation of one or more high-grade pathogens in a blood culture specimen or the identification of a common skin contaminant or skin flora in at least two separate blood culture specimens from different sites in the same patient; and (ii)* susceptibility to antibiotic treatment*. Exclusion criteria were presence of signs of infection or being in incubation period at admission, referral from another hospital department or health center, and age under 14 yrs.

The Acute Physiology and Chronic Health Evaluation (APACHE) II score [26] was determined for each patient, considering the worst reading in the first 24 h of ICU stay, in order to evaluate the severity of illness and calculate the predicted mortality rate.

### 2.4. Antibiotic Selection Criteria

Antibiotic prescription was structured in three levels, with the aim of treating patients in the shortest time possible with the most appropriate antibiotic according to the infection focus and clinical situation. The first level was the implementation of an empiric antibiotic treatment in patients with clinical suspicion of infection. For this purpose, ICU physicians had access via the touch screen in the unit to the LRMs, which depicted the percentage activity of different antibiotics against the microorganisms usually detected in each infectious process, allowing them the possibility of prescribing the treatment in accordance with these data (guidelines). In the second action level, after the putative isolation or identification of one or more microorganisms in a sample from the patient, the physician received a PMRTR prepared by the microbiology specialists (see above). Finally, after identification of the bacteria, the microbiology laboratory issued a definitive report with the corresponding antibiogram.

The study was conducted under the following conditions: (i) the information provided by these GERB applications was not binding in any case. Physicians were not obliged to use the LRM and/or PMRTR guidelines and could base their selection of antibiotic therapy on exclusively clinical criteria (in accordance with the guidelines of the Hospital Infections Committee); (ii) the selection of antibiotic treatment was always adapted to the clinical situation of patients and took account of any therapeutic limitations, including allergies, drug interactions, and toxicity, considering renal and hepatic function, administration routes, dose, dose intervals, and so forth; (iii) in the LRM and/or PMRTR guidelines, an antibiotic was recommended when active against ≥75% of all microorganisms isolated in the same infection focus during the previous 12 months; (iv) broad-spectrum antibiotics were used for severe infections (especially in low respiratory tract infections and bacteremias); (v) after receipt of the PMRTR, the physician was able to modify or maintain the initial empiric treatment; and (vi) after receipt of the definitive microbiological report, with bacterial identification and corresponding antibiogram, deescalation was conducted when indicated, selecting the most appropriate antibiotic(s) according to clinical, microbiological, and pharmacological criteria. No study was made of the reasons for the therapeutic decisions taken by the physicians in this study.

Antibiotic treatment was considered appropriate when at least one of the prescribed antibiotics was active* in vitro* against the isolated microorganism(s) and the drug regimen was in accordance with current medical standards. The appropriateness of the therapeutic option and/or antibiotic prescription was assessed by comparing each of the antibiotics recommended and/or prescribed in a patient with the definitive antibiogram of the microorganism(s) finally identified as the causal agent, when available. An antibiotic with synergic activity, for example, aminoglycosides, was not considered appropriate when it was the only antibiotic active against the isolate* in vitro*.

### 2.5. Data Collection

Data were gathered in all studied patients on admission date, sex, age, main diagnosis, personal history of interest (allergies, other diseases, previous medication, etc.), chronic organ failure (hepatic, renal, pulmonary, cardiovascular, and immunosuppression, as defined by APACHE II), clinical progress during hospital stay using a semiquantitative scale [27], analytical results (full blood count, biochemistry, cultures, etc.), and daily body temperature. Other variables recorded were the empiric antibiotic treatment selected (indicating whether LRM guidelines were followed or not), any treatment change (indicating whether PMRTR recommendation was followed), the dose, dosing frequency, administration route, possible toxicity, total hospital stay, and date of discharge or death. Patients were followed up until their death or ICU discharge.

### 2.6. Statistical Analysis

SPSS 17.0 for Windows was used for the data analyses. Pearson's chi-square test (with continuity correction when required) was used to compare the appropriateness of prescribed antibiotic treatments according to the application of clinical criteria, LRM guidelines, or PMRTR recommendations and to compare patient mortality rates in each of these situations and when no empiric treatment was administered. Fisher's exact test in a 2 × 2 tables was used when the sample size was too small and conditions for Pearson's chi-square test application were not met. The Student's* t*-test was employed to compare the mean days of ICU stay as a function of the criteria used for empiric antibiotic treatment prescription (clinical, LRM, or PMRTR) and the receipt or not empiric treatment. The Mann-Whitney* U* test was used when the distribution of a variable was nonnormal according to the results of a previously applied Shapiro-Wilk test. *P* < 0.05 was considered significant in all tests.

## 3. Results

Between October 2008 and December 2010, 218 patients in the ICU of our hospital met the study eligibility criteria, 139 males (63.8%) and 79 females (36.2%). The mean APACHE II score of the study cohort was 16.9 ± 7.5 (range, 2–40).

Microbiological documentation of infection was obtained in 137 patients (62.8%) (Table 1), with the identification of 262 different microorganisms from 185 respiratory samples, 26 urine samples, and 51 blood cultures (without considering duplicates in the same sample type). Gram-negative bacteria (63.7%) were the most frequent, followed by Gram-positive bacteria (30.2%), fungi (5.0%), and anaerobes (1.1%). A single microorganism was isolated in 68 (49.6%) of the patients (43 microorganisms in respiratory samples, 2 in urine samples, and 23 in blood cultures), while multiple microorganisms were isolated from the same or different samples in the remaining 69 patients. Microorganisms were isolated from respiratory samples alone in 74 patients, from blood cultures alone in 24 patients, and from urine samples alone in 2 patients; in the remaining 37 patients, microorganisms were isolated from two or more samples from different infection foci. No microorganisms were isolated in culture in 81 (37.2%) of the patients, whose clinical suspicion of infection was not microbiologically confirmed.

### 3.1. Assessment of Appropriateness of Antibiotic Prescriptions that Follow LRM Guidelines

Empiric antibiotic treatment was implemented for suspicion of nosocomial infection in 173 of the 218 study patients (79.4%), but LRM guidelines were only followed in 44 of these (25.4%) (Table 2). When clinical criteria lone were adopted, the most frequently prescribed antibiotics were amoxicillin-clavulanic acid, vancomycin, levofloxacin, carbapenems (meropenem or imipenem), and ceftriaxone. When LRM guidelines were followed, the most frequently antibiotics prescribed were carbapenems, vancomycin, piperacillin-tazobactam, amikacin, and linezolid. After sample culture, microorganisms were isolated in 77 of the 129 patients (59.7%) prescribed according to clinical criteria and in 15 (34.1%) of the 44 patients prescribed in accordance with LRM guidelines.

The empiric treatment was a single antibiotic in 76 (43.9%) of the 173 patients (amoxicillin-clavulanic acid in 43 [56.6%] of cases); two antibiotics in 47 (27.2%) of the 173 patients, three in 41 (23.7%), and four in 9 (5.2%). Monotherapy was prescribed in 51.8% of patients treated according to clinical criteria (amoxicillin-clavulanic acid in 64.2% of cases)* versus* 20.5% of those treated according to LRM. The appropriateness of the empiric antibiotic treatments was evaluated by analyzing the antibiotics prescribed in the 92 patients for whom an antibiogram of the isolated microorganism was available. In the 77 of these patients treated according to clinical criteria, 36.4% of the antibiotics prescribed to this group proved to be active against the isolated bacteria, in comparison to 80.0% of the 15 patients treated according to LRM guidelines. Hence, the percentage appropriateness of the empiric antibiotic treatment was significantly higher (*P* = 0.005) when LRM guidelines were followed.

### 3.2. Assessment of Appropriateness of PMRTR Recommendations

The microbiology laboratory issued 139 PMRTRs for 96 (44.0%) of the 218 patients in the study, with a total of 362 recommendations for antibiotic therapy (Table 2). When Gram-negative bacilli were isolated in culture, the most frequently recommended antibiotics were imipenem, amikacin, and piperacillin-tazobactam; when Gram-positive cocci in clusters were isolated, they were linezolid and vancomycin, and when Gram-positive cocci in chains were isolated, they were vancomycin and levofloxacin. The appropriateness of PMRTR therapeutic recommendations was evaluated by comparing each of the 362 recommended antibiotics with the definitive antibiogram of the microorganism(s) eventually identified in each patient, which showed that 90.3% of recommended antibiotics were active against the identified bacterium/bacteria.

### 3.3. Assessment of Appropriateness of Antibiotic Prescription after Receipt of PMRTR Recommendations

Antibiotic treatment prescription recommendations were followed in 68 (70.8%) of the 96 patients for whom a PMRTR was issued, leading to the modification of initial empiric treatment in 36 patients (52.9%), its maintenance in 4 patients (5.9%), or the commencement of treatment in 28 previously untreated patients (41.2%). Clinical criteria rather than the received PMRTR were followed in 19 patients (19.8%). In the remaining 9 patients (9.4%), PMRTRs were issued after ICU discharge or death.

Table 2 shows that when the criteria were exclusively clinical, the most frequently prescribed antibiotics were ceftriaxone, tobramycin, carbapenems, and vancomycin, in this order. However, when PMRTR recommendations were followed, the most frequent were carbapenems, amikacin, vancomycin, piperacillin-tazobactam, and linezolid. Combined therapy with two antibiotics was predominant both in the prescriptions following clinical criteria (mainly ceftriaxone plus tobramycin) and in those following PMRTR (mainly carbapenem plus amikacin).

The appropriateness of antibiotic prescriptions after PMRTR receipt was assessed by comparing the antibiotic prescribed to each patient with the definitive antibiogram of the microorganism(s) finally identified in each sample. According to the definitive antibiogram, 42.1% of the antibiotics prescribed following clinical criteria were active against the isolated bacteria, whereas 82.4% of those prescribed in accordance with PMRTR guidelines were active. The percentage appropriateness of antibiotic treatment prescription was therefore significantly higher (*P* = 0.001) when PMRTR was followed.

### 3.4. Global Assessment of GERB Use

#### 3.4.1. GERB Use and Level of Associated Appropriateness

As noted above, LRMs were followed for the prescription of empiric antibiotic therapy in 44 (20.2%) of the 218 patients in the study and were not followed in 174 (79.8%) patients. Active antibiotics against the isolated bacteria were prescribed in 80.0% of the former group but in only 36.4% of the latter. Out of the 87 patients with available PMRTR, the recommendations were followed in 68 (78.2%) but not in 21 (21.8%) patients. Active antibiotics were prescribed against isolated bacteria in 82.4% of the former cases but in only 42.1% of the latter.

LRM and PMRTR were both followed in only 8 (9.2%) of the 87 patients for whom they were both available (LRM for empiric treatment prescription, then PMRTR for modification of the initial treatment). Solely clinical criteria were adopted in 32 (36.8%) of the patients, while either LRM or PMRTR were followed in the remaining 47 (54.0%).

#### 3.4.2. Mortality and Hospital Stay

The influence of GERB (LRM and PMRTR) was evaluated on the two main clinical variables: mortality and days of ICU stay. This analysis only included the 137 patients with diagnostic certainty of infection, that is, when a clinically significant microorganism was isolated.

The mean ICU stay of patients who received empiric treatment following LRM guidelines was 13.8 days, with a mortality rate of 20.0%; their mean ICU admission APACHE II score was 17.7. The mean ICU stay of patients who received empiric treatment following clinical criteria was 19.5 days, with a mortality rate of 27.3%; their mean ICU admission APACHE II score was 17.6. There were no significant differences between patients receiving empiric treatment according to LRM guidelines or clinical criteria in mortality (*P* = 0.751) or days of stay (*P* = 0.156), even when nonsurvivors were included in the analysis (*P* = 0.519).

The mean ICU stay of patients treated according to PMRTR recommendations was 19.7 days, with a mortality of 29.4%; their mean ICU admission APACHE II score was 18.0. The mean ICU stay of patients treated according to clinical criteria was 20.1, with a mortality of 36.8%; their mean ICU admission APACHE II score was 19.0. There were no significant differences between patients treated according to PMRTR recommendations or clinical criteria in mortality (*P* = 0.735) or days of stay (*P* = 0.943), even when nonsurvivors were included (*P* = 0.219).

## 4. Discussion

Physicians should prescribe an appropriate empiric antibiotic treatment in patients with clinical suspicion of infection. The criteria adopted are usually based on their own experience or on guidelines that are often developed in another setting, even in another country. Hence, therapeutic treatment is frequently not adapted to the microbial epidemiology of the specific healthcare area, which may favor therapeutic failure. Numerous publications by scientific societies and healthcare institutions have emphasized the need to consider local epidemiology in the development of therapeutic guidelines for the prescription of empiric antibiotics [15–17, 21, 22].

Computerized decision support systems (CDSS) are clinical consultation systems that assist physicians in diagnostic and therapeutic decision making by analyzing patient and population data. They have proven effective to improve medical care, reduce prescription errors, and enhance compliance with recommendations [28, 29]. These programs do not replace clinical judgment but rather increase the information available for physicians to be able to make correct decisions [22]. A systematic review associated successful CDSS implementation with the integration of the system in the clinical process and with the availability of recommendations at the time and place of decision making [30].

Evans et al. [28] evaluated the effects of a computerized anti-infective-management program for real-time patient-specific recommendations on the type of antimicrobial, dose, administration route, and treatment duration, finding it to be useful in surgical prophylaxis and in targeted and empiric treatments; use of this program also significantly reduced the number of days that patients received antimicrobial treatment in the ICU. Thursky et al. [31] associated the utilization of a real-time microbiology browser and CDSS for antibiotic prescription with a reduction in total antibiotic prescriptions, especially in the most widely prescribed broad-spectrum antibiotics.

In the present study, GERB-derived LRMs permitted the rates of bacterial resistance to antibiotics to be monitored, based on the information in the LIS of the microbiology laboratory. In general, this application permits (i) structuring of epidemiologic data by hospital area and by infectious disease, (ii) daily and automatic updating with new laboratory results, (iii) presentation of the information in a web environment, and (iv) presentation in readily interpreted graphics of data on bacterial resistance to the antibiotics habitually used in the treatment of a given infectious disease. After positive cultures are obtained, PMRTRs provide a preliminary report on the putative identification of isolated microorganism(s), issuing therapeutic recommendations based on their most likely susceptibility profile according to the local epidemiology of the hospital unit and the specific infectious disease in question.

According to the present results, the utilization of LRMs and PMRTRs contributed to the adaptation of antibiotic treatments, favoring the administration of the most active antibiotics in clinical situation. The lower percentage appropriateness of empiric antibiotic treatments that followed clinical criteria was related to the prescription of monotherapy antibiotics, especially amoxicillin-clavulanic acid, or to the use of combinations of narrow-spectrum antibiotics with high microorganism resistance rates. The considerable increase in percentage appropriateness with treatments following LRM and/or PMRTR guidelines was associated with the prescription of antibiotics with very low resistance rates.

A major challenge in evaluating expert systems that support therapeutic decision making concerns the adherence of physicians to their use, which was relatively low in the present study, especially in relation to LRM guidelines. Physicians may be reluctant to abandon their own criteria or well-established antimicrobial therapy guidelines with recognized prestige, especially in the prescription of empiric treatments [32]. The much higher adherence to PMRTR may be attributable to its provision of an explicit recommendation in a printed report with the signature of a microbiology specialist. Adherence to the GERB applications was stronger when the clinical situation of the patient was more severe, finding a mean APACHE II score of 21 in the eight patients for whom LRM and PMRTR were followed, or when the recommendation was to continue with the same antibiotic therapy.

The results of this study did not support the hypothesis that application of these GERB applications would significantly reduce the mortality rate and length of ICU stay. Previous studies also found no significant reduction in mortality after the development and implementation of local treatment protocols, although these were associated with an improvement in empiric therapy adaptation and a reduction in the antibiotic treatment duration [13, 25].

In common with other investigations of measures designed to improve antibiotic use, it was not possible to conduct a randomized controlled trial, and the design of our prospective study was therefore quasi-experimental. Patients were not managed with a specific protocol, and it was therefore not possible to control for all relevant clinical variables. We cannot rule out the influence of unmeasured variables and we did not evaluate the response to antibiotic therapy according to predefined clinical variables. A further limitation was the difficulty in assessing the clinical impact of the GERB applications, because no microorganism was isolated in a large percentage (37.2%) of patients; therefore, although there was suspicion of infection, there was no microbiological confirmation. It is likely that a large number of the empiric treatments, following either clinical criteria or LRM, were not for a true bacterial infection, although the early onset of antibiotic treatment may possibly have avoided growth of the microorganism in culture. The selection of one antibiotic or another would not have determined the final outcome in the first situation but may have done so in the second. In fact, it is possible that the lower number of patients in which a given microorganism was isolated when empiric treatment was based on LRM guidelines (34.1%) is related to the high appropriateness rates for antibiotic prescriptions in line with these guidelines. Finally, our assessment of the appropriateness of antibiotic treatments did not consider the isolation of other microorganisms against which these treatments are not active. This is the case of fungi, such as* Candida* spp., which only represented 5% of the microorganisms identified.

In conclusion, these new GERB applications offer dynamic, highly accessible, and easily interpreted instruments to assist physicians in the selection of antibiotic treatment. Their implementation increases the percentage of patients administered with an appropriate initial empiric therapy. It would be of interest to perform a similar study in different hospital departments over the same time period in order to examine variations among them.

## Figures and Tables

**Table 1:** Distribution by sample type of the 262 microorganisms isolated in the 137 patients.

Microorganism	Respiratory samples	Urine	Blood cultures	Total
*Acinetobacter baumannii *	2	1	3	**6**
*Bacteroides fragilis *			3	**3**
*Candida albicans *	4	5		**9**
*Candida parapsilosis *		1	1	**2**
*Candida tropicalis *			2	**2**
*Citrobacter koseri *	5		1	**6**
*Enterobacter aerogenes *	4		1	**5**
*Enterobacter cloacae *	10			**10**
*Enterobacter sakazakii *	1			**1**
*Enterococcus faecalis *	2		7	**9**
*Enterococcus faecium *	1		1	**2**
*Escherichia coli *	22	7	2	**31**
*Haemophilus influenza *	8			**8**
*Klebsiella oxytoca *	4			**4**
*Klebsiella pneumoniae *	18	3		**21**
*Morganella morganii *	1			**1**
*Proteus mirabilis *	7	3	1	**11**
*Pseudomonas aeruginosa *	29	5	5	**39**
*Pseudomonas stutzeri *	1			**1**
*Serratia liquefaciens *		1		**1**
*Serratia marcescens *	7		2	**9**
*Serratia plymuthica *	1			**1**
*Staphylococcus aureus *	32		6	**38**
*Staphylococcus epidermidis *			10	**10**
*Staphylococcus hominis *			4	**4**
*Stenotrophomonas maltophilia *	12			**12**
*Streptococcus *grupo* viridans *			2	**2**
*Streptococcus pneumoniae *	14			**14**

**Table 2:** Distribution of the antibiotics administered to the 173 patients receiving empiric treatment of the antibiotics recommended by PMRTR (362 recommendations) and of the antibiotics administered to the 87 patients whose treatment was modified after PMRTR emission.

Antibiotic	Patients who received empirical treatment (173 patients)		Antibiotic recommended by PMRTR (362 recommendations)			Treatment modified after PMRTR emission (87 patients)	
	Empirical treatment according to clinical criteria (129 patients)	Empirical treatment according to LRM (44 patients)	When Gram-negative bacillus was isolated	When Gram-positive cocci in clusters were isolated	When Gram-positive cocci in chains were isolated	Treatment modified according to clinical criteria but not PMRTR (19 patients)	Treatment modified according to PMRTR (68 patients)
Amoxicillin-clavulanic acid	52 (22.3%)	1 (1.0%)	2 (0.8%)	—	—	3 (7.9%)	1 (0.8%)
Piperacillin-tazobactam	6 (2.6%)	15 (15.6%)	61 (23.6%)	—	—	—	17 (12.9%)
Cefazolin	7 (3.0%)	2 (2.1%)	—	—	—	1 (2.6%)	—
Ceftriaxone	24 (10.4%)	2 (2.1%)	2 (0.8%)	—	—	6 (15.8%)	3 (2.3%)
Cefotaxime	8 (3.4%)	1 (1.0%)	13 (5.0%)	—	7 (15.6%)	1 (2.6%)	7 (5.3%)
Ceftazidime	5 (2.1%)	1 (1.0%)	5 (1.9%)	—	—	2 (5.3%)	3 (2.3%)
Cefepime	1 (0.4%)	1 (1.0%)	13 (5.0%)	—	—	—	2 (1.5%)
Imipenem	10 (4.3%)	7 (7.3%)	68 (26.4%)	—	8 (17.8%)	2 (5.3%)	14 (10.6%)
Meropenem	15 (6.4%)	12 (12.6%)	7 (2.7%)	—	—	3 (7.9%)	16 (12.1%)
Levofloxacin	25 (10.7%)	5 (5.2%)	20 (7.8%)	—	10 (22.2%)	2 (5.3%)	7 (5.3%)
Ciprofloxacin	3 (1.3%)	—	1 (0.4%)	—	—	—	—
Amikacin	14 (6.0%)	15 (15.6%)	61 (23.6%)	—	—	3 (7.9%)	26 (19.6%)
Tobramycin	12 (5.2%)	5 (5.2%)	2 (0.8%)	—	—	6 (15.8%)	3 (2.3%)
Gentamicin	2 (0.9%)	—	—	—	—	2 (5.3%)	—
Vancomycin	28 (12.0%)	18 (18.8%)	—	27 (45.8%)	10 (22.2%)	4 (10.4%)	23 (17.4%)
Linezolid	10 (4.3%)	6 (6.3%)	—	28 (47.5%)	2 (4.4%)	2 (5.3%)	10 (7.6%)
Other	11 (4.7%)	5 (5.2%)	3 (1.2%)	4 (6.7%)	8 (17.8%)	1 (2.6%)	—
Total	**233 (100%)**	**96 (100%)**	**258 (100%)**	**59 (100%)**	**45 (100%)**	**38 (100%)**	**132 (100%)**

LRM: local resistance map; PMRTR: preliminary microbiological reports with therapeutic recommendation.
